# A commentary on “Is opening-wedge high tibial osteotomy superior to closing-wedge high tibial osteotomy in treatment of unicompartmental osteoarthritis? A meta-analysis of randomized controlled trials” [Int J Surg 60 (2018) 153–163]

**DOI:** 10.1097/JS9.0000000000000856

**Published:** 2023-10-26

**Authors:** Hongfeng Ding, Jiangnan Zhang, Chang Jiang

**Affiliations:** Department of Orthopedic Surgery, The First People’s Hospital of Wenling, Wenzhou Medical University Affiliated Wenling Hospital, Wenling, Zhejiang, People’s Republic of China

*Dear Editors*,

Knee osteoarthritis (OA) is one of the most common joint disorders, and it causes considerable pain and immobility. Malalignment increases the risk of progression of OA of the knee^1^. For patients with OA of the medial compartment of the knee, a valgus high tibial osteotomy (HTO) is a treatment option^2,3^. HTO may be indicated in association with meniscal transplantation, cartilage repair procedures, and knee ligament reconstruction^4^. There are ongoing discussions regarding the choice of method for preoperative planning, the choice of osteotomy site, and the choice of operative technique. Alterations in joint line angles, patellar height, posterior tibial slope, leg length, and correction accuracy are among the controversial issues. The techniques most commonly used include closed-wedge osteotomy (CWO) and open-wedge osteotomy (OWO), stabilized by a locking plate^5,6^. Each technique has its advantages and disadvantages^7,8^. Currently, whether CWO or OWO is better in OA remains controversial in published clinical trials. We have great pleasure of reading the article titled ʻIs opening-wedge high tibial osteotomy superior to closing-wedge high tibial osteotomy in treatment of unicompartmental osteoarthritis? A meta-analysis of randomized controlled trialsʼ published by Wang *et al*.^9^ They suggested that there were no significant differences between OWO and CWO regarding the VAS knee pain scores, HSS knee scores, walking distances, or hip-knee-ankle (HKA) angles. Furthermore, there were no significant differences between the two groups in complication and survival rates. Nevertheless, there was a significantly greater tibial slope angle in OWO patients. Therefore, they were unable to conclude which method is superior. Further high-quality RCTs with longer follow-up times should be conducted to draw a more definitive conclusion. At the outset, we would like to congratulate the authors for writing an informative article with novelty. However, we have several suggestions and comments that we would like to communicate with the authors.

First, several flaws in the study design and statistical methods are worth pointing out. The study protocol registration is a key element of a systematic review and it is considered to be a reliable approach not only to enhance and maintain the clarity of a successful review, but also to reduce the risk of selective reporting bias^10^. However, the authors did not register the study protocol in the International Prospective Register of Systematic Reviews (PROSPERO). The authors showed that they searched the relevant studies in electronic databases, including PubMed, Embase, Web of Science, Cochrane Library, and Google databases. Since only 599 patients were included in this meta-analysis, the results would be more convincing if the authors had included other databases such as Medline, NLM Gateway, BIOSIS previews, and Clinicaltrials.gov to obtain more studies, with less chances of published articles being missed. Besides, there was some mistakes in the search string: (ʻOpenʼ) and (ʻClosedʼ OR ʻClosingʼ) and (ʻOsteotomyʼ OR ʻTibialʼ). This should be (ʻOpenʼ) and (ʻClosedʼ OR ʻClosingʼ) and (ʻOsteotomyʼ AND ʻTibialʼ) and (ʻOsteoarthritisʼ). Essential published articles can be missed if the manual search protocol is incomplete.

Second, there were some mistakes in their table of general characteristics of the included studies. The sample size of the two studies published by Magyar *et al*.^11,12^ was not correct, which should be ʻ25 vs. 25ʼ and ʻ19 vs. 16ʼ. Moreover, many of the included studies were from Netherlands, which may cause selective bias (country bias). The figure of the risk of bias assessment summary and risk of bias graph also have some mistakes. We have carefully reviewed the included studies and evaluated their risks of bias according to the Cochrane tool, we found some of our results to be inconsistent with the results of the authors. Our results were shown in Figure 1 and Figure 2, most of the included studies did not report the blinding of participants and blinding of outcome assessment.

**Figure 1:**
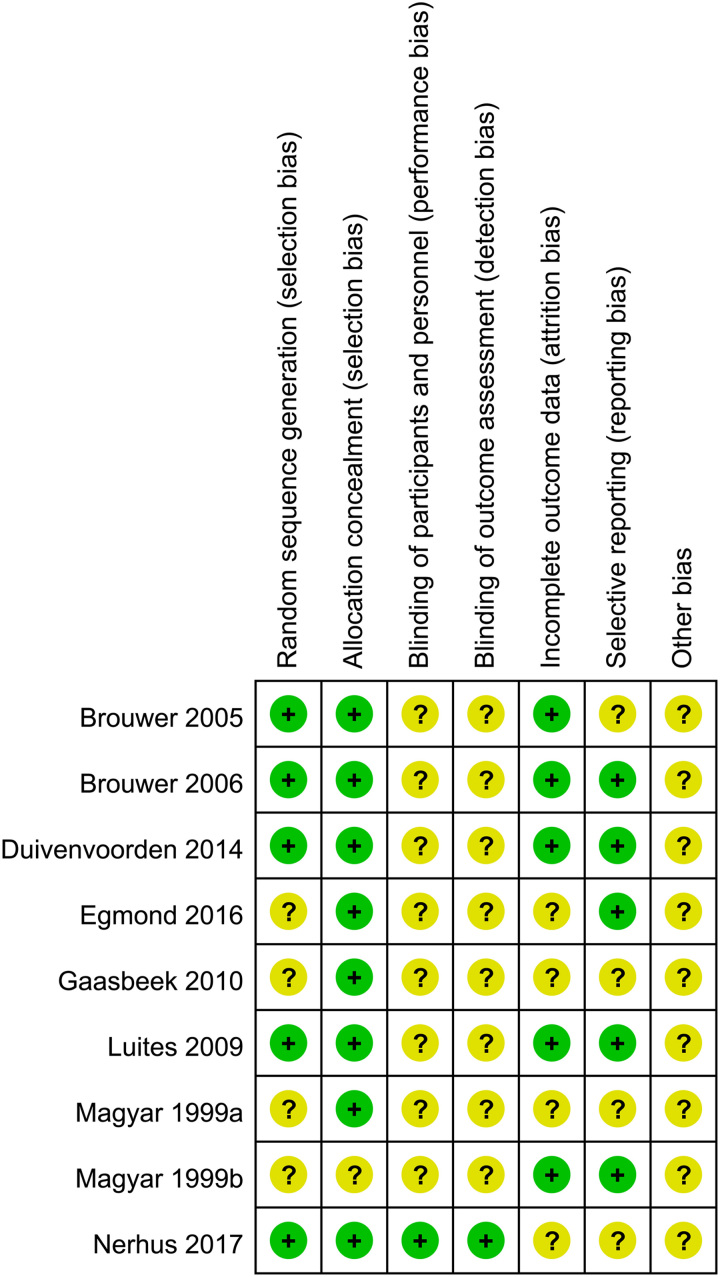
The risk of bias of the included randomized controlled trials.

**Figure 2:**
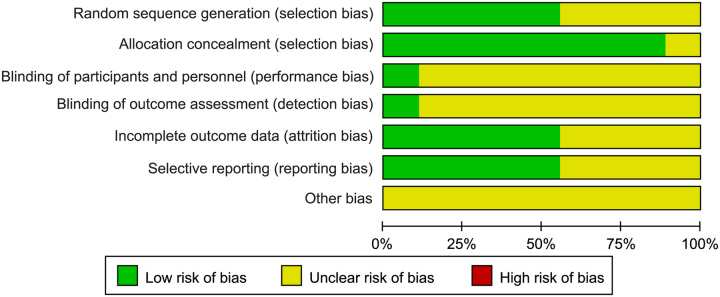
The risk of bias graph.

Third, a high degree of heterogeneity should not lead to definitive conclusions as based on the Cochrane Handbook for Systematic Reviews^13^ and high heterogeneity can reduce the reliability of the conclusion. We found significant heterogeneity existed in the outcome VAS knee pain at greater than 5 years, HKA angle at less than 5 years, HKA angle at greater than 5 years, tibial slope angle, and complication rate. With such large heterogeneities in some of these outcomes, subgroup analysis, and sensitivity analysis should be conducted. The authors performed subgroup analysis for VAS knee pain stratified by risk of bias (low or unclear/high), effect-model (fixed or random effect-model), follow-up duration (≤2 years or>2 years) and fixation method (puddu plate or others). However, the effect-model is not a confounding factor, should be removed out. Also, the outcome HKA angle at less than 5 years existed high heterogeneity (*I*^2^=51.9%) with seven studies included, the authors did not perform subgroup analysis for it. Besides, the authors stated ʻA sensitivity analysis was performed to identify the source of the heterogeneityʼ in their statistical analysis section. However, no figures about sensitivity analysis were found in their meta-analysis. A more robust model, the Inverse Variance Heterogeneity (IVhet) model, has been introduced in the study by Doi *et al*.^14^ They examined an improved alternative to the random effects (RE) model for meta-analysis of heterogeneous studies.

Therefore, we suggest the authors use the IVhet model to re-analyze the outcomes of this study.

Fourth, we are very suspicious of the authenticity of the extractive data. In regard to the outcome of VAS knee pain at less than 5 years, we carefully reviewed the included studies, we found some difference from the authors’ data: Luites *et al*.^15^ and two studies of Magyar *et al*.^11,12^ did not report the VAS knee pain score. As for the outcome HSS knee score, Egmond *et al*.^16^ used Knee Society Score (KSS) not HSS knee score, Magyar *et al*.^12^ only reported the HSS with mean and range, SD was not found, how did the authors pool this data? The same concern also existed in the outcome HKA angle at less than 5 years: two studies of Magyar *et al*.^11,12^ only reported the HKA with mean, range, SD was also not found.

Based on the above issues, we extracted the data from the included studies and pool them to analyze. As shown in Figure 3, the pooled results showed that no significant difference was found in the OWO group and the CWO group regarding VAS knee pain at less than 5 years (WMD=0.390; 95% CI: −0.178–0.957; *P*=0.178; heterogeneity *χ*^2^=2.44, df=3; *I*^2^=0%, *P*=0.486). Moreover, no significant difference was found in the OWO group and the CWO group regarding HKA angle at less than 5 years (WMD=0.466; 95% CI: −0.594–1.526; *P*=0.389; heterogeneity *χ*^2^=11.28, df=4; *I*^2^=64.5%, *P*=0.024, Fig. 4). With high heterogeneities found in the outcome HKA angle at less than 5 years, sensitivity analysis was conducted to detect the source of heterogeneity. As shown in Figure 5, the results of the sensitivity analysis showed that a significant effect was observed after excluding any one single study, suggesting that the results were relatively robust. Moreover, we recently evaluated the potential publication bias of HKA angle at less than 5 years through a funnel plot, Begg and Egger test (Fig. 6). The funnel plot and *P* values from Egger’s tests indicated that publication bias was found (*P*=0.041).

**Figure 3:**
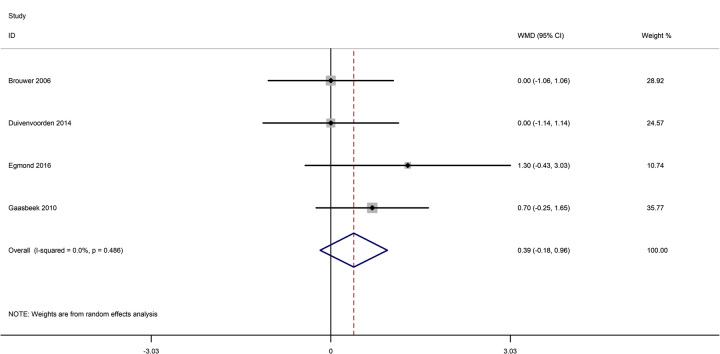
Forest plots of the included studies comparing the VAS knee pain at < 5 years.

**Figure 4:**
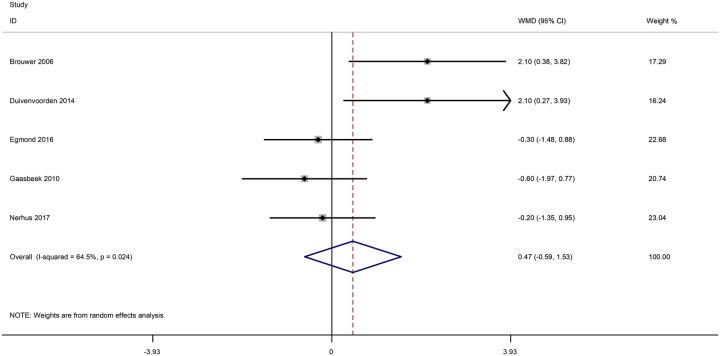
Forest plots of the included studies comparing the HKA angle at < 5 years.

**Figure 5:**
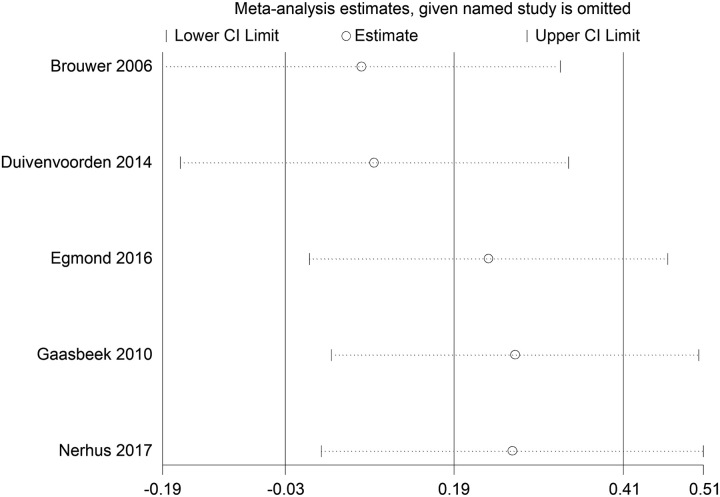
Sensitivity analysis of HKA angle <5 years.

**Figure 6:**
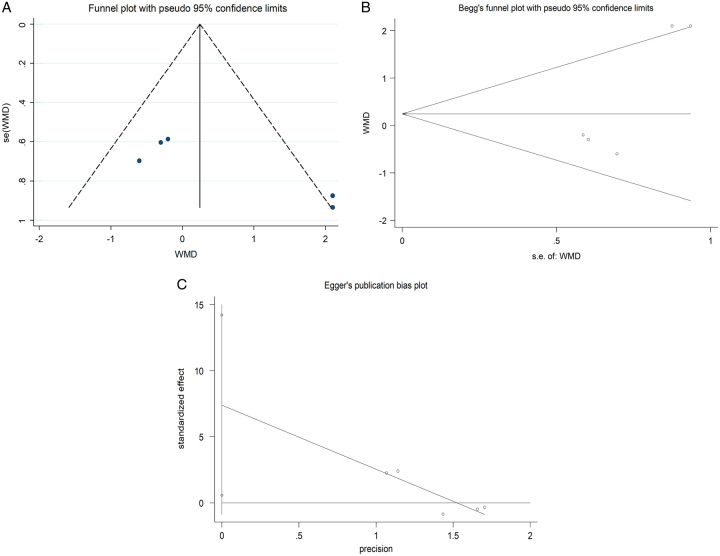
Funnel plot (A), Begg's test (B) and Egger's test (C) of the HKA angle <5 years.

Finally, the quality assessment of evidence according to the GRADE criteria^17^ was not reported in this study. We recently evaluated the quality of the evidence of the included studies using GRADE criteria by GRADE Profiler 3.6 version. The results were demonstrated in Table 1. As shown in Table 1, most of the outcomes were graded as ʻvery lowʼ or ʻlowʼ because of the risk of bias and inconsistency. Therefore, the authors should be extremely cautious when drawing conclusions.

**Table 1:** The GRADE evidence quality for each outcome.

Quality assessment							No of patients		Effect			
No of studies	Design	Risk of bias	Inconsistency	Indirectness	Imprecision	Other considerations	Opening-wedge high tibial osteotomy versus closing-wedge high tibial osteotomy	Control	Relative (95% CI)	Absolute	Quality	Importance
VAS knee pain at <5 years (Better indicated by lower values)												
4	Randomised trials	Very serious^a^^b^	No serious inconsistency	No serious indirectness	No serious imprecision	None	124	135	–	MD 0.39 higher (0.18 lower to 0.96 higher)	⊕⊕ΟΟ LOW	Important
VAS knee pain at >5 years (Better indicated by lower values)												
2	Randomised trials	Very serious^a^^b^	Serious^c^	No serious indirectness	No serious imprecision	None	54	63	–	MD 0.28 higher (1.58 lower to 2.14 higher)	⊕ΟΟΟ Very low	Important
HKA angle at <5 years (Better indicated by lower values)												
5	Randomised trials	Very serious^a^^b^	Serious^c^	No serious indirectness	No serious imprecision	Reporting bias^d^	159	170	–	MD 0.47 higher (0.59 lower to 1.53 higher)	⊕ΟΟΟ Very low	Critical
HKA angle at >5 years (Better indicated by lower values)												
2	Randomised trials	Very serious^a^^b^	Serious^c^	No serious indirectness	No serious imprecision	None	54	63	–	MD 0.38 higher (0.85 lower to 1.6 higher)	⊕ΟΟΟ Very low	Critical
Tibial slope angle (Better indicated by lower values)												
3	Randomised trials	Serious^b^	Serious^c^	No serious indirectness	No serious imprecision	None	79	77	–	MD 4.93 higher (3.72 to 6.15 higher)	⊕⊕ΟΟ Low	Important
Walking distance (Better indicated by lower values)												
2	Randomised trials	Serious^b^	No serious inconsistency	No serious indirectness	No serious imprecision	None	81	92	–	MD 0.7 higher (0.51 lower to 1.91 higher)	⊕⊕⊕Ο Moderate	Important
Complications												
3	Randomised trials	Very serious^a^^b^	Very serious^c^	No serious indirectness	No serious imprecision	None	40/115 (34.8%)	25/119 (21%)	RR 1.66 (1.08 to 2.54)	139 more per 1000 (from 17 more to 324 more)	⊕ΟΟΟ Very Low	Important
								24%		158 more per 1000 (from 19 more to 370 more)		

GRADE Working Group grades of evidence: High-quality: Further research is very unlikely to change our confidence in the estimate of effect. Moderate quality: Further research is likely to have an important impact on our confidence in the estimate of effect and may change the estimate. Low quality: Further research is very likely to have an important impact on our confidence in the estimate of effect and is likely to change the estimate. Very low quality: We are very uncertain about the estimate.

a Some of the included studies did not report random sequence generation.

b Most of the included studies did not report the blinding of participants and blinding of outcome assessment.

c Significant heterogeneity (*I*^2^>50% or *P*<0.1) was found.

d Publication bias was found.

We thank Wang *et al*. again for their meaningful work in summarizing the evidence of OWO and CWO in the treatment of unicompartmental OA. However, a more clear and meticulous methodology, an exhaustive search strategy, and a scrutinized analysis of the data provided could have enhanced the robustness and accuracy of the findings and reinforced the clinical impact of this extensive literature review and meta-analysis.

## Ethical approval

Not applicable.

## Sources of funding

None.

## Author contribution

H.D.: statistical analysis and writing; J.Z.: statistical analysis and literature search; C.J.: study design.

## Conflicts of interest disclosure

The authors declare that they have no competing interests.

## Research registration unique identifying number (UIN)

Not applicable.

## Guarantor

Chang Jiang.

## Provenance and peer review

Commentary, internally reviewed.
